# Case of Strumous Diathesis

**Published:** 1858-07

**Authors:** S. L. Gill

**Affiliations:** Campbell Terrace, Bow.


### ARTICLE XIII.

*Case of Strumous Diathesis.* Communicated by Dr. S. L. Gill, Campbell Terrace, Bow.

Caroline C., Bow Common, Middlesex, aged thirty-six, of strumous diathesis, applied to me, having suffered for three years from a running sore on the right side of the sternum over the cartilage of the fourth rib.

There was great inability to open the mouth, as the side of the jaw was much thickened, and the tonsil of the same side considerably enlarged, presenting a very suspicious appearance, accompanied by a most offensive discharge. Several cicatrices under the angle of the jaw on the affected side, indicated points from which pus had been evacuated from glandular abscesses. The glands about and under the angle of the jaw and side of the neck were much enlarged, as was also the carotid, whilst the side of the face in front of the ear was prominent and hard. The lymphatics were much corded, and the sterno-mastoid muscle puckered in with the skin, the sternal end of the clavicle was likewise thickened; in fact, taking the case as a whole, my first impression was unfavorable. The mouth was in a decidedly unwholesome condition, and the teeth covered with tartar.

Whilst making my examination of the enlarged glands, a thin pus exuded from the opening over the rib, and on passing a very fine gold probe into the opening, it traversed high up in the course of the sterno-mastoid muscle. The dens sapientiæ was decayed, and considering it would at least cause irritation, I removed it, when a small quantity of pus exuded. I ordered unguent. potass. iodidi. to be applied over the glandular swellings, and cod liver oil internally. In five weeks I had the satisfaction to see the case cured; the sinus over the rib had healed, and the inability to open the jaw was greatly improved; in fact, I may say she was restored to health. S. L. Gill.

P. S. The enlargement upon the tonsil, I excised.—*Lon. Quar. Jour. Den. Sci.*

